# 1,25-dihydroxyvitamin D_3_ Protects against Macrophage-Induced Activation of NFκB and MAPK Signalling and Chemokine Release in Human Adipocytes

**DOI:** 10.1371/journal.pone.0061707

**Published:** 2013-04-24

**Authors:** Cherlyn Ding, John P. H. Wilding, Chen Bing

**Affiliations:** Obesity Biology Research Unit, Department of Obesity and Endocrinology, Institute of Ageing and Chronic Disease, University of Liverpool, Liverpool, United Kingdom; University of Warwick – Medical School, United Kingdom

## Abstract

Increased accumulation of macrophages in adipose tissue in obesity is linked to low-grade chronic inflammation, and associated with features of metabolic syndrome. Vitamin D_3_ may have immunoregulatory effects and reduce adipose tissue inflammation, although the molecular mechanisms remain to be established. This study investigated the effects of vitamin D_3_ on macrophage-elicited inflammatory responses in cultured human adipocytes, particularly the signalling pathways involved. Macrophage-conditioned (MC) medium (25% with adipocyte maintenance media) markedly inhibited protein expression of the nuclear factor-κB (NFκB) subunit inhibitor κBα (IκBα) (71%, *P*<0.001) and increased NFκB p65 (1.5-fold, *P* = 0.026) compared with controls. Treatment with 1,25-dihydroxyvitamin D_3_ (1,25(OH)_2_D_3_) abolished macrophage-induced activation of NFκB signalling by increasing IκBα expression (2.7-fold, *P* = 0.005) and reducing NFκB p65 phosphorylation (68%; *P*<0.001). The mitogen-activated protein kinase (MAPK) signalling was activated by MC medium, which was also blunted by 1,25(OH)_2_D_3_ with a downregulation of phosphorylated p38 MAPK (32%, *P* = 0.005) and phosphorylated Erk1/2 (49%, *P* = 0.001). Furthermore, MC medium (12.5% or 25%) dose-dependently upregulated secretion of key proinflammatory chemokines/cytokines (22-368-fold; all *P*<0.001) and this was significantly decreased by 1,25(OH)_2_D_3_: IL-8 (61% and 31%, *P*<0.001), MCP-1 (37%, *P*<0.001 and 36%, *P* = 0.002), RANTES (78% and 62%, *P*<0.001) and IL-6 (29%, *P*<0.001 and 34%, *P* = 0.019). Monocyte migration-elicited by adipocytes treated with 1,25(OH)_2_D_3_ was also reduced (up to 25%, *P*<0.001). In conclusion, vitamin D_3_ could be anti-inflammatory in adipose tissue, decreasing macrophage-induced release of chemokines and cytokines by adipocytes and the chemotaxis of monocytes. Our data suggests these effects are mediated by inhibition of the NFκB and MAPK signalling pathways.

## Introduction

Growing evidence suggests that vitamin D_3_ has pleiotropic functions, beyond its well established roles in bone and mineral metabolism, particularly with regards to insulin secretion and action [1], [2]. Vitamin D_3_ deficiency may contribute to the pathogenesis of a number of disorders, including obesity and metabolic syndrome [3], [4], [5]. Epidemiological studies and clinical trials have shown that obese individuals tend to have low vitamin D_3_ status [6], [7], [8]. Although the mechanisms are not clear, sequestration of vitamin D by adipose tissue, less exposure to sunlight and low intake of vitamin D in obese individuals may contribute [8], [9], [10]. 25-hydroxycholecalciferol (25(OH)D_3_) is the major circulating form of vitamin D_3_, which is converted to the active form 1,25-dihydroxycholecalciferol (1,25(OH)_2_D_3_). 1,25(OH)_2_D_3_ acts as a ligand for the vitamin D receptor (VDR) that facilitates the transcription of target genes [11], [12]. Interestingly, recent studies demonstrate the presence of VDR and vitamin D-metabolizing enzymes in human adipose tissue [13], [14]. Therefore, human adipose tissue could be a direct target of vitamin D_3_, and deficiency may have pathological consequences in this tissue [15].

With adipose tissue expansion in obesity, there is a marked increase in the synthesis and release of proinflammatory factors (e.g. TNFα, IL-6, IL-8 and MCP-1), and this may contribute to the elevated circulating levels seen as well as to local tissue inflammation [16], [17]. Adipose tissue inflammation, exacerbated by increased infiltration of macrophages and other immune cells, is a central pathological process of adipose tissue dysfunction in obesity [18], [19]. Recent work from our group and others has demonstrated that macrophage-derived factors potently stimulate the release of proinflammatory chemokines/cytokines and a number of proteins involved in extracellular matrix remodelling from human preadipocytes and adipocytes; these factors are known to induce inflammation, fibrosis and insulin resistance in adipose tissue, which is associated with metabolic disorders [20], [21], [22], [23]. Evidence has accumulated that vitamin D_3_ exerts potent immunoregulatory effects, such as inhibiting the production of TNFα, IL-6 and IL-8 by peripheral blood mononuclear cells in humans [24], [25], [26]. The effects of vitamin D_3_ may be through targeting the nuclear factor-kB (NFkB) and mitogen-activated protein kinase (MAPK) signalling pathways [27], [28], [29], [30]. The emerging role of adipose tissue in adaptive immunity has raised the question whether vitamin D_3_ could protect against adipose tissue inflammation.

Studies in murine 3T3-L1 adipocytes have produced inconsistent results, 1,25(OH)_2_D_3_ being reported to increase or decrease gene expression of IL-6 and MCP-1 [31], [32], [33]. Information concerning vitamin D_3_ action in human adipose tissue is scarce. Recent studies from our group and others have shown that 1,25(OH)_2_D_3_ decreased cytokine-induced expression and release of MCP-1 by human preadipocytes and mature adipocytes [34], [35]. However, the mechanisms and the extent to which vitamin D_3_ modulates inflammation in human adipose tissue, especially in macrophage-adipocyte crosstalk, remains to be established. These studies were therefore conducted to investigate the effect of 1,25(OH)_2_D_3_ on macrophage-induced inflammatory responses in human adipocytes. The molecular mechanisms particularly the NFκB and MAPK signalling pathways and the downstream effects of vitamin D_3_ were also studied.

## Materials and Methods

### Adipocyte Cell Culture

Human preadipocytes derived from subcutaneous adipose tissue of a female Caucasian subject (BMI 21; age 44 years) were purchased from PromoCell (Heidelberg, Germany). Cells were seeded at 40,000/cm^2^ and grown in 6-well or 24-well plates in preadipocyte growth medium, containing DMEM-Ham’s F-12 (1∶1, vol/vol) and supplemented with 100 U/ml penicillin, 100 µg/ml streptomycin, and 0.25 µg/ml amphotericin B (Lonza, Twekesbury, UK), at 37°C in a humidified atmosphere of O_2_:CO_2_ (95∶5%). At confluence, cells were induced to differentiate at day 0 by incubation for 3 days in Dulbecco's Modified Eagle's Medium (DMEM) and Ham's F12 (in a 1∶1 ratio) medium containing 32 µM biotin, 1 µM dexamethasone, 200 µM 3-isobutyl-1-methyl-xanthine, 100 nM insulin, 11 nM L-Thyroxine (all from Sigma, Poole, Dorset, UK), 8 µM Rosiglitazone (GlaxoSmithKline, Uxbridge, UK), 100 U/ml penicillin, 100 µg/ml streptomycin, and 0.25 µg/ml amphotericin B. After induction, cells were cultured in maintenance medium containing 3% foetal calf serum (FCS; Sigma), 100 nM insulin, 32 µM biotin and 1 µM dexamethasone until fully differentiated. Differentiation into mature adipocytes was visualised under the microscope by observing the accumulation of lipid droplets.

### Macrophage-conditioned Medium

Human THP-1 myelomonocytic cell line was purchased from Health Protection Agency Culture Collections (Porton Down, Salisbury UK). THP-1 monocytes (1×10^6^ cells/ml) were cultured in a 150 cm^2^ flask in Roswell Park Memorial Institute (RPMI-1640) medium (containing 10% FCS, 100 U/ml penicillin and 100 µg/ml streptomycin) at 37°C in a humidified atmosphere of O^2^:CO^2^ (95∶5%). For the preparation of macrophage-conditioned (MC) medium, THP-1 monocytes were differentiated into macrophages with 100 nM phorbol 12-myristate 13-acetate (PMA) (Sigma) for 48 h. The medium was replaced with PMA-free and FCS-free RPMI-1640 medium for 24 h; this medium was collected, filtered through a 0.22 µm filter and stored at −80°C for later use.

### Cell Treatment

To examine the effect of vitamin D_3_ on basal levels of NFκB and MAPK signalling, adipocytes (at day 11 post-differentiation) were treated with 1,25(OH)_2_D_3_ (10^−11^ and 10^−8^ M) (ENZO Life Sciences, Plymouth Meeting, PA, USA) for 24 h, and another group of adipocytes received no treatment as controls. To further assess whether vitamin D_3_ reduces macrophage-induced inflammatory response, adipocytes were pretreated with vitamin D_3_ (10^−11^ and 10^−8^ M) for 48 h and then exposed to the MC medium (12.5% or 25% in adipocyte maintenance media), in the presence or absence of 1,25(OH)_2_D_3_ (10^−11^ and 10^−8^ M) for a further 4 h, 6 h or 24 h. Separate groups of cells were treated with the RPMI medium (12.5% or 25% in adipocyte maintenance media) for the same period as controls. At the end of each experiment, cells and the culture media were collected and stored at −80°C until analysis.

To evaluate the effect vitamin D_3_ on the migration of monocytes, adipocytes were treated with vitamin D_3_ (10^−11^ and 10^−8^ M) or without (control) for 24 h; the culture media was then collected for performing the chemotaxis assay.

### Western Blotting

Western blotting was performed as previously described [21]. Briefly, total cellular protein was obtained using lysis buffer (50 mM Tris-HCl, pH 6.7, 10% Glycerol, 4% SDS, 2% 2-mercaptoethanol) with freshly added protease inhibitor cocktail and phosphatase inhibitor cocktail (both from Sigma). Protein concentrations were determined by the BCA method. Protein samples (40 µg/lane) were separated on 10% Tricine-SDS polyacrylamide slab gels (Mini Protean Tetra, Bio-Rad, Hemel Hempstead, UK) and transferred to nitrocellulose membranes (Hybond-C Extra, Amersham Bioscience, UK) by wet transfer (Trans Blot, Bio-Rad). The successful transfer of proteins to the membranes was assessed by Ponceau S staining.

For immunodetection, the membranes were blocked for 1 hour at room temperature in Tris-buffered saline (TBS) containing 5% BSA and 0.1% Tween-20. The membranes were then incubated with the primary antibody, including Ικβα (New England Biolabs Ltd, Hitchin, Hertfordshire, UK), phosphorylated NFκB p65 (Sigma) and phosphorylated p38 MAPK and phosphorylated Erk1/2 (both from New England Biolabs Ltd, Hitchin, Hertfordshire, UK), at 1∶1000 dilution at 4°C overnight. Subsequently, membranes were washed in PBS with 0.1% Tween-20 and then incubated with a HRP-conjugated secondary antibody (Bio-Rad, Hertfordshire, UK or Cell Signalling, Danvers, MA, US). Signals were detected by chemiluminescence using a SuperSignal West Pico Chemiluminescent Substrate (Pierce, Rockford, IL, US). The intensity of signals was evaluated using the Molecular Imager ChemiDoc XRS+ System (Bio-Rad). The size of the protein bands was estimated with PageRuler protein markers (Fermentas, York, UK). The membranes were further probed with GAPDH (Abcam, Cambridge, UK) or total Akt (Cell Signalling) as a loading control. The results were normalised to the value of GAPDH or total Akt.

### Real-time PCR

Total RNA was extracted from cells using Trizol (Invitrogen, Paisley, UK). For reverse transcription, 0.5 µg of total RNA was converted to first-strand cDNA in a volume of 10 µl reaction using an iScript first strand synthesis kit (Bio-Rad), which was then diluted at 1∶4. Real-time PCR was carried out in a final volume of 12.5 µl, containing 1 µl cDNA (equivalent to 10 ng of RNA), optimized concentrations of primers, TaqMan probe (FAM-TAMRA) and a master mix made from qPCR core kit (Eurogentec, Seraing, Belgium) using a Stratagene Mx3005P instrument. The sequences of primer and probe used for human IL-8, MCP-1, RANTES (regulated on activation, normal T cell expressed and secreted), IL-1β, IL-6 and β-actin were as described previously [22], [36]. PCR reactions were performed in duplicate and the PCR amplification was initiated at 95°C for 10 min, followed by 40 cycles (95°C for 15 sec and 60°C for 1 min). Non-template controls were run in parallel. All Ct values were within the range of 20–33 cycles. The results were normalised to the house-keeping gene β-actin values and expressed as fold changes of Ct value relative to controls using the 2^−ΔΔct^ formula.

### Enzyme-Linked Immunosorbent Assay

Protein release of IL-8, MCP-1, RANTES and IL-6 by adipocytes, and by THP-1 macrophages were measured as protein concentrations in cell culture medium, using DuoSet ELISA Development kits (R&D Systems, Abingdon, UK).

### Transmigration Assay

THP-1 monocytes at a density of 2×10^6^ cells/ml were suspended in RPMI-1640 and 100 µl of monocyte suspension was added to the upper chamber of QCM™ chemotaxis transwells (Fisher Scientific, Loughborough, UK) with a pore size of 5 µm. 150 µl of adipocyte culture medium, harvested from the cells treated with vitamin D_3_ (10^−8^ M) or without (control) for 24 h, was added to the lower chamber of transwells. After incubation for 4 h at 37°C in a humidified atmosphere of 5% CO_2_ and 95% air, the number of monocytes that migrated to the lower chamber of transwells was determined using the MTT assay with a cell density standard curve.

### LDH Assay

Adipocyte viability following various treatments was assessed as the release of lactate dehydrogenase (LDH) into the cell culture medium, using a colourimetric cytotoxicity detection kit (Roche Diagnostics GmbH, Mannheim, Germany). LDH levels were measured by a spectrophotometer at 492 nm with a reference wavelength of 620 nm at room temperature.

### Statistical Analysis

Results are presented as means ± SEM. Comparison of means between two groups was analysed using Student’s t-test. Comparison among more than two groups was performed by one-way ANOVA coupled with Bonferroni’s *t*-test. Differences were considered as statistically significant at *P*<0.05.

## Results

### 1,25-dihydroxyvitamin D_3_ Inhibits Macrophage-Induced Activation of NFκB

**Figure 1:**
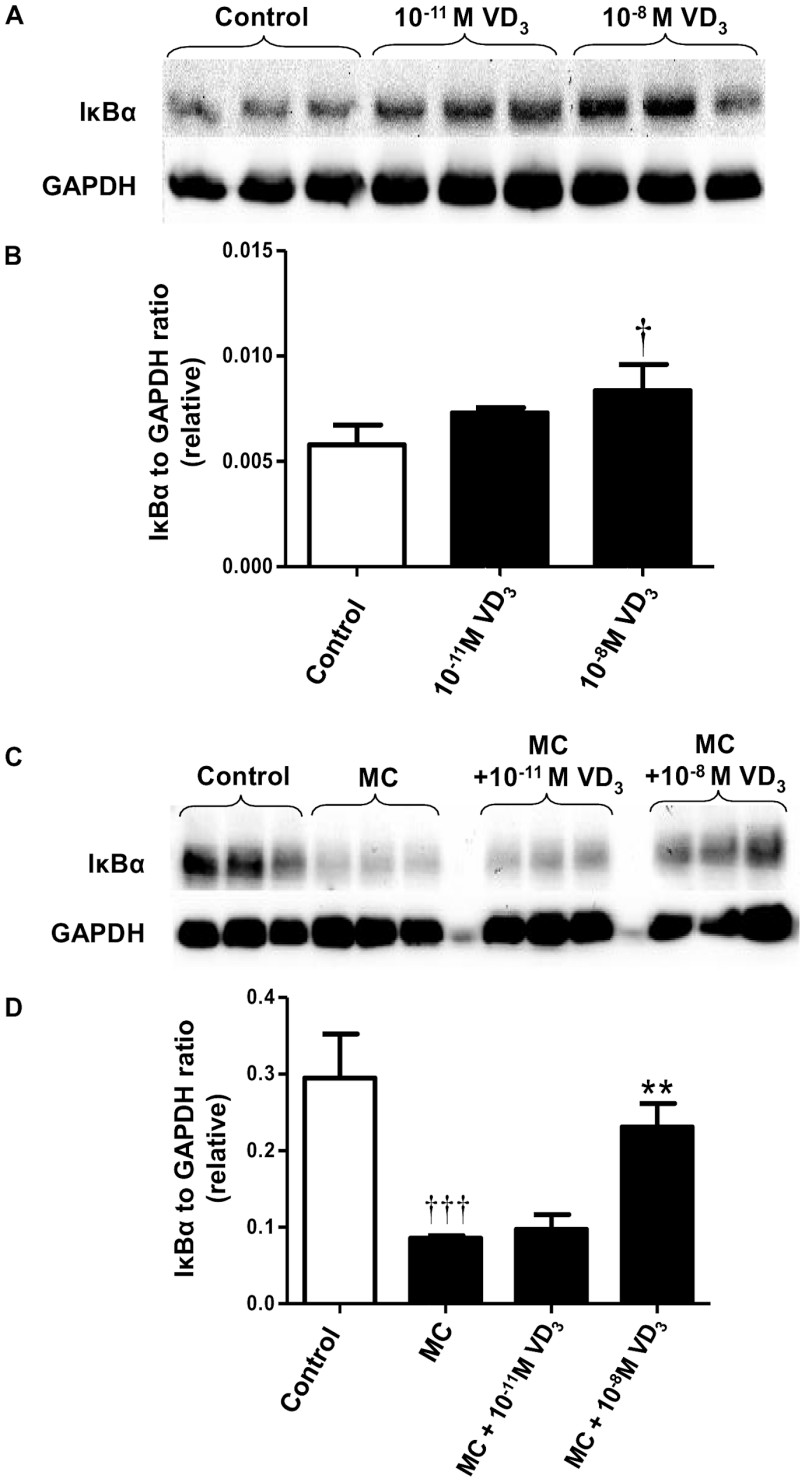
Effects of 1,25-dihydroxyvitamin on protein abundance of IκBα in human adipocytes. Effect of 1,25(OH)_2_D_3_ on basal level of IκBα was studied in adipocytes incubated with vitamin D_3_ (10^−11^ M and 10^−8^ M) or without (control) for 72 h. (A) Phosphorylated IκBα protein content in cell lysates was analysed by western blotting, with GAPDH used as loading controls. (B) Signals were quantified by densitometry. Effect of 1,25(OH)_2_D_3_ on MC medium-induced phosphorylation of IκBα was studied in adipocytes pretreated with 1,25(OH)_2_D_3_ (10^−11^ M and 10^−8^ M), followed by incubation with RPMI-1640 medium (control) or macrophage conditioned (MC) medium (25%) for another 24 h. Protein expression of phosphorylated IκBα in cell lysates was analysed by western blotting. (C) Representative western blots. (D) Signals were quantified by densitometry. Data are means ± SEM, normalised to GAPDH levels, n = 3 per group. ^†^ *P*<0.05, ^†††^ *P*<0.001 vs controls; **P<0.01 vs MC medium. The results were confirmed by three independent experiments.

As the activation of NFκB signalling pathway has a key role in the signal transduction of proinflammatory chemokines/cytokines, we first assessed whether vitamin D_3_ affects basal and MC medium-stimulated protein expression of NFκB subunits IκBα and NFκB p65 by human adipocytes. As shown in Fig. 1A–B, a low dose of 1,25(OH)_2_D_3_ (10^−11^ M) had no effect on IκBα while a higher dose (10^−8^ M) of 1,25(OH)_2_D_3_ significantly increased basal IκBα protein abundance (by 1.4-fold, *P*  = 0.042). Exposure to MC medium led to a marked reduction in IκBα protein abundance in adipocytes (by 71%, *P*<0.001) compared with controls (Fig. 1C–D). Although the lower dose of 1,25(OH)_2_D_3_ (10^−11^ M) did not reverse this reduction, 1,25(OH)_2_D_3_ at higher dose (10^−8^ M) abolished the inhibitory effect of the MC medium, leading to a 2.7-fold increase (*P*  = 0.005) in IκBα levels compared with the MC group (Figure 1C–D).

**Figure 2:**
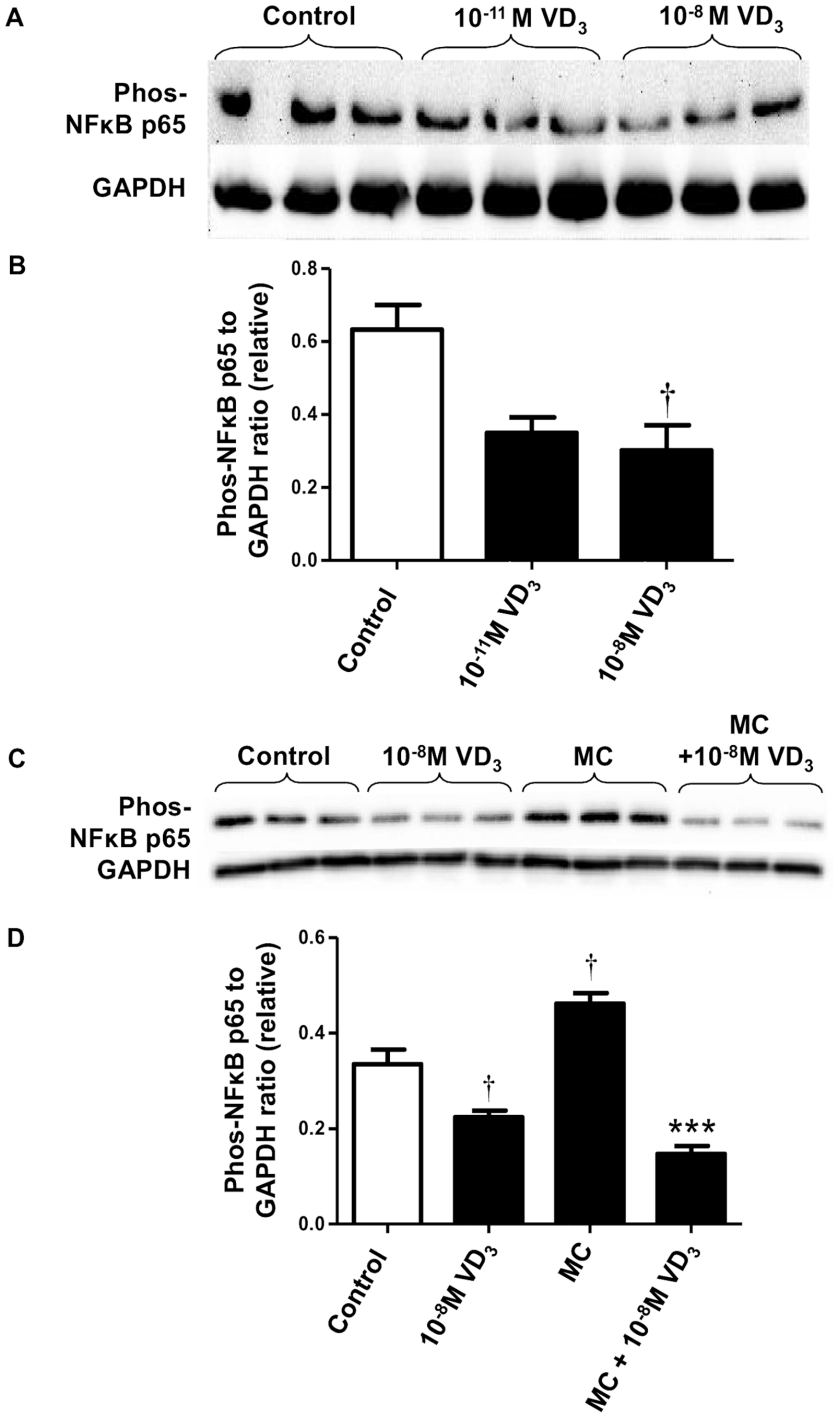
1,25-dihydroxyvitamin D_3_ inhibits MC medium-induced phosphorylation of NFκB p65 in human adipocytes. Effect of 1,25(OH)_2_D_3_ on basal level of NFκB p65 was studied in adipocytes incubated with vitamin D_3_ (10^−11^ M and 10^−8^ M) or without (control) for 72 h. (A) Phosphorylated NFκB p65 protein content in cell lysates was analysed by western blotting, with GAPDH used as loading controls. (B) Signals were quantified by densitometry. Effect of 1,25(OH)_2_D_3_ on MC medium-induced phosphorylation of NFκB p65 was studied in adipocytes pretreated with 1,25(OH)_2_D_3_ (10^−8^ M), followed by incubation with RPMI-1640 medium (control) or macrophage conditioned (MC) medium (25%) for another 24 h. Protein expression of phosphorylated NFκB p65 in cell lysates was analysed by western blotting. (C) Representative western blots. (D) Signals were quantified by densitometry. Data are means ± SEM, normalised to GAPDH levels, n = 3 per group. ^†^ *P*<0.05 vs controls; ***P<0.001 vs MC group. The results were confirmed by three independent experiments.

For NFκB p65, treatment with 1,25(OH)_2_D_3_ at both doses (10^−11^ and 10^−8^ M) significantly reduced basal protein abundance of phosphorylated NFκB p65 by 45% (*P*  = 0.05) and 52% (*P*  = 0.026), respectively (Fig. 2A–B). Upon MC medium stimulation, there was a significant increase in phosphorylated NFκB p65 compared with controls (by 1.4-fold, *P*  = 0.021) (Fig. 2C–D). However, this upregulation was completely blunted by the treatment with 1,25(OH)_2_D_3_ (10^−8^ M) (*P*<0.001) (Fig. 2C–D).

### 1,25-dihydroxyvitamin D_3_ Inhibits Macrophage-Induced Activation of MAPK

**Figure 3:**
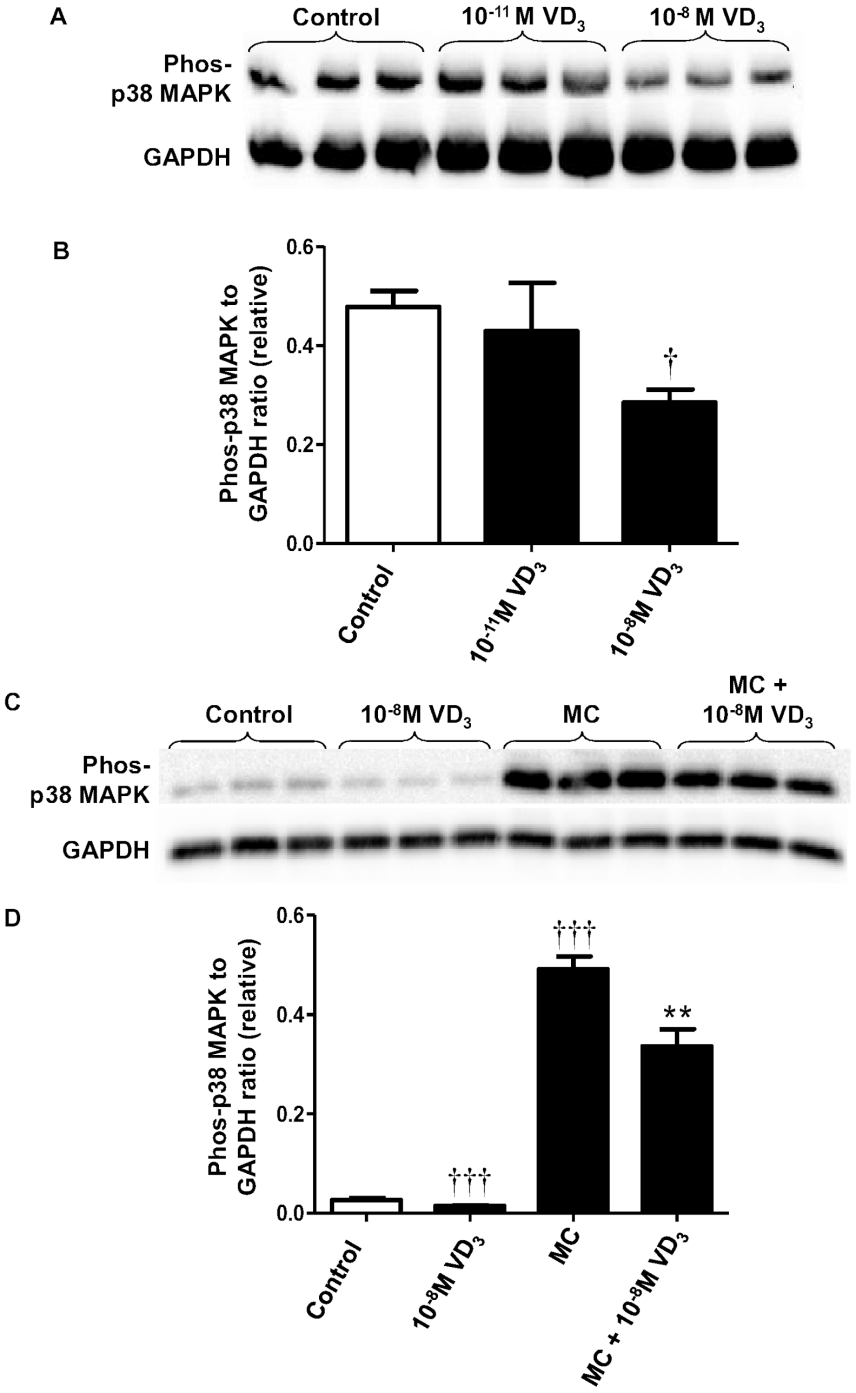
1,25-dihydroxyvitamin D_3_ reduces MC medium-induced phosphorylation of p38 MAPK in human adipocytes. Effect of 1,25(OH)_2_D_3_ on basal level of p38 MAPK was studied in adipocytes incubated with vitamin D_3_ (10^−11^ M and 10^−8^ M) or without (control) for 72 h. (A) Phosphorylated p38 MAPK protein content in cell lysates was analysed by western blotting, with GAPDH used as loading controls. (B) Signals were quantified by densitometry. Effect of 1,25(OH)_2_D_3_ on MC medium-induced phosphorylation of p38 MAPK was studied in adipocytes pretreated with 1,25(OH)_2_D_3_ (10^−8^ M), followed by incubation with RPMI-1640 medium (control) or macrophage conditioned (MC) medium (25%) for another 6 h. Protein expression of phosphorylated p38 MAPK in cell lysates was analysed by western blotting. (C) Representative western blots. (D) Signals were quantified by densitometry. Data are means ± SEM, normalised to GAPDH levels, n = 3 per group. ^†^ *P*<0.05, ^†††^ *P*<0.001 vs controls; **P<0.01 vs MC group. The results were confirmed by three independent experiments.

**Figure 4:**
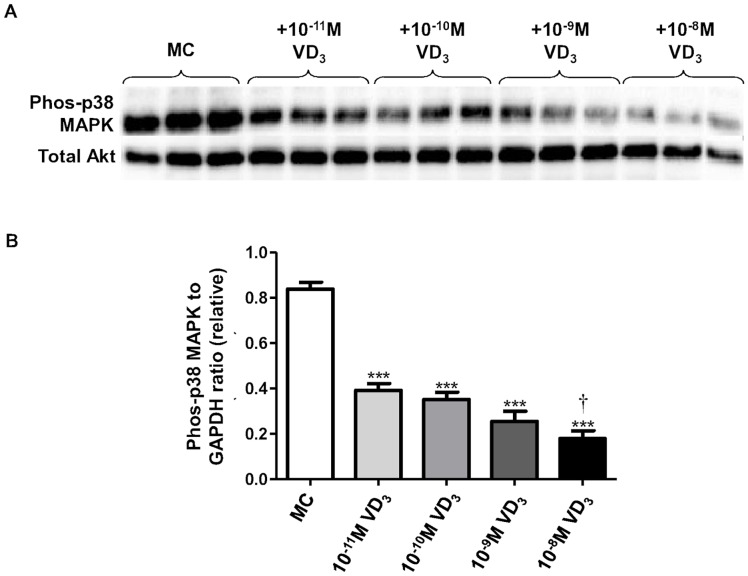
1,25-dihydroxyvitamin D_3_ dose dependently decreases MC medium-induced phosphorylation of p38 MAPK in human adipocytes. Adipocytes were incubated with increasing doses of 1,25(OH)_2_D_3_ (10^−11^ M, 10^−10^ M, 10^−9^ M, 10^−8^ M) or without (control) for 72 h, followed by stimulation with macrophage conditioned (MC) medium (25%) for another 24 h. Protein expression of phosphorylated p38 MAPK in cell lysates was analysed by western blotting, with GAPDH used as loading controls. (A) Representative western blots. (B) Signals were quantified by densitometry; data are means ± SEM, normalised to total Akt levels, n = 3 per group. ^†^ *P*<0.05 vs 10^−11^ M dose group, ***P<0.001 vs MC group.

Basal protein abundance of phosphorylated p38 MAPK was decreased by the treatment with higher dose (10^−8^ M) of 1,25(OH)_2_D_3_ (by 34%, *P*  = 0.025) (Fig. 3A–B) although it was unaffected by the lower dose (10^−11^ M). In adipocytes stimulated with the MC medium, protein abundance of phosphorylated p38 MAPK was highly induced (by 18-fold; *P*<0.001) compared with controls (Fig. 3C–D). Vitamin D_3_ (10^−8^ M) significantly reduced the induction of phosphorylated p38 MAPK by the MC medium (by 32%, *P*  = 0.005) (Fig. 3C–D). Furthermore, the inhibitory effect of vitamin D_3_ on p38 MAPK appears to be dose-dependent; treatment with 1,25(OH)_2_D_3_ at doses ranging from 10^−11^ M to 10^−8^ M led to a significant reduction in p38 MAPK (from 50% to 80%, all *P*<0.001) (Fig. 4A–B).

**Figure 5:**
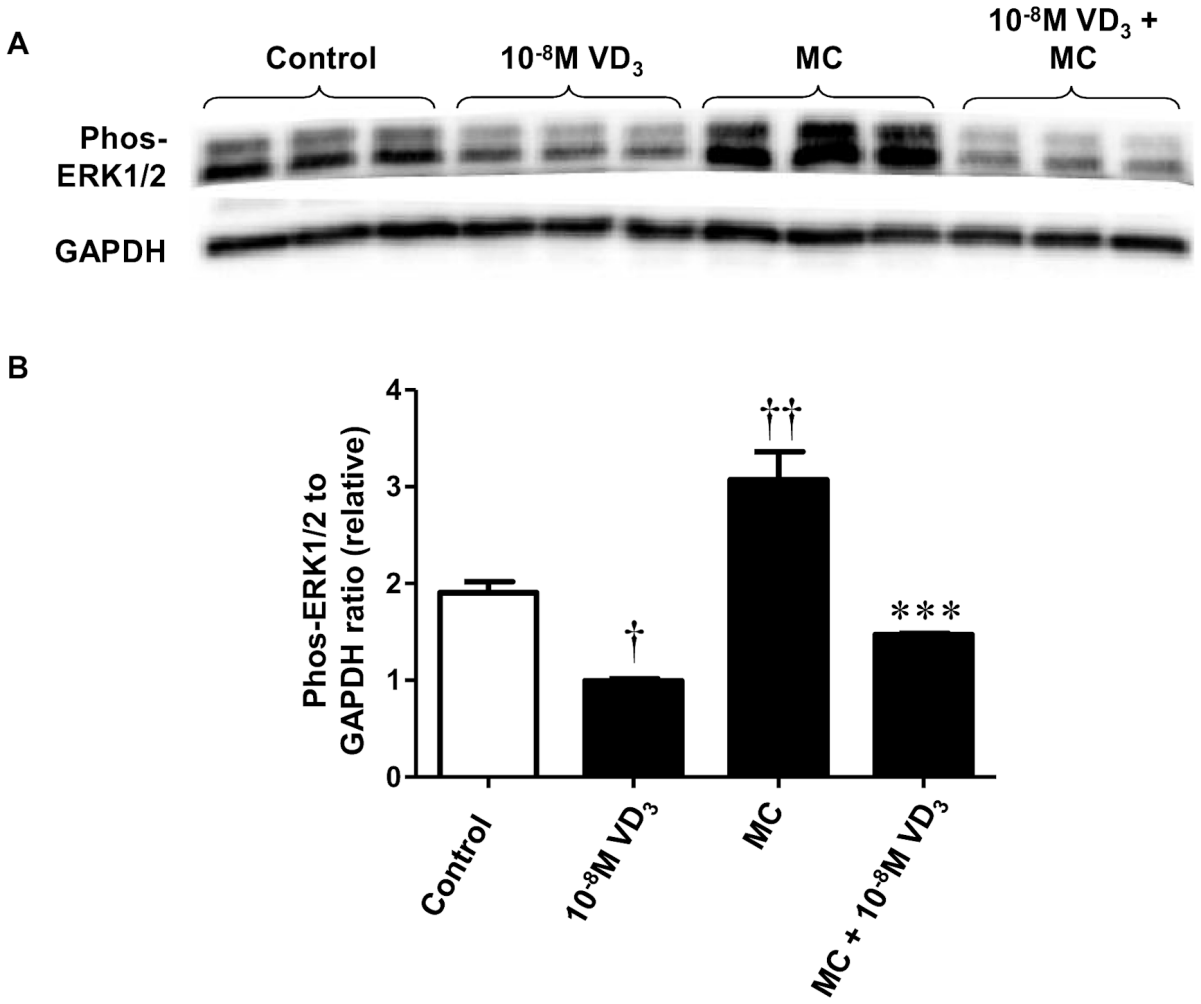
1,25-dihydroxyvitamin D_3_ attenuates MC medium-induced phosphorylation of Erk1/2 in human adipocytes. Adipocytes were pretreated with 1,25(OH)_2_D_3_ (10^−8^ M) or without for 48 h, followed by incubation with RPMI-1640 medium (control) or macrophage conditioned (MC) medium (25%) for another 24 h. Protein expression of phosphorylated Erk1/2 in cell lysates was analysed by western blotting, with GAPDH used as loading controls. (A) Representative western blots. (B) Signals were quantified by densitometry; data are means ± SEM, normalised to GAPDH levels, n = 3 per group. ^††^ *P*<0.01 vs controls; ***P<0.01 vs MC group. The results were confirmed by three independent experiments.

In addition to inhibiting p38 MAPK, treatment with 1,25(OH)_2_D_3_ (10^−8^ M) significantly reduced basal protein abundance of phosphorylated Erk1/2 (by 48%, *P*  = 0.02). As shown in Fig. 5A–B, when adipocytes were stimulated by MC medium, there was a marked upregulation in protein expression of phosphorylated Erk1/2 compared with controls (by 1.6-fold, *P*  = 0.004). However, this increase was completely abolished by 1,25(OH)_2_D_3_ (10^−8^ M) (*P*  = 0.001) (Fig. 5A–B).

### 1,25-dihydroxyvitamin D_3_ Decreases Macrophage-Stimulated Production of the Chemokines/Cytokines by Human Adipocytes

**Figure 6:**
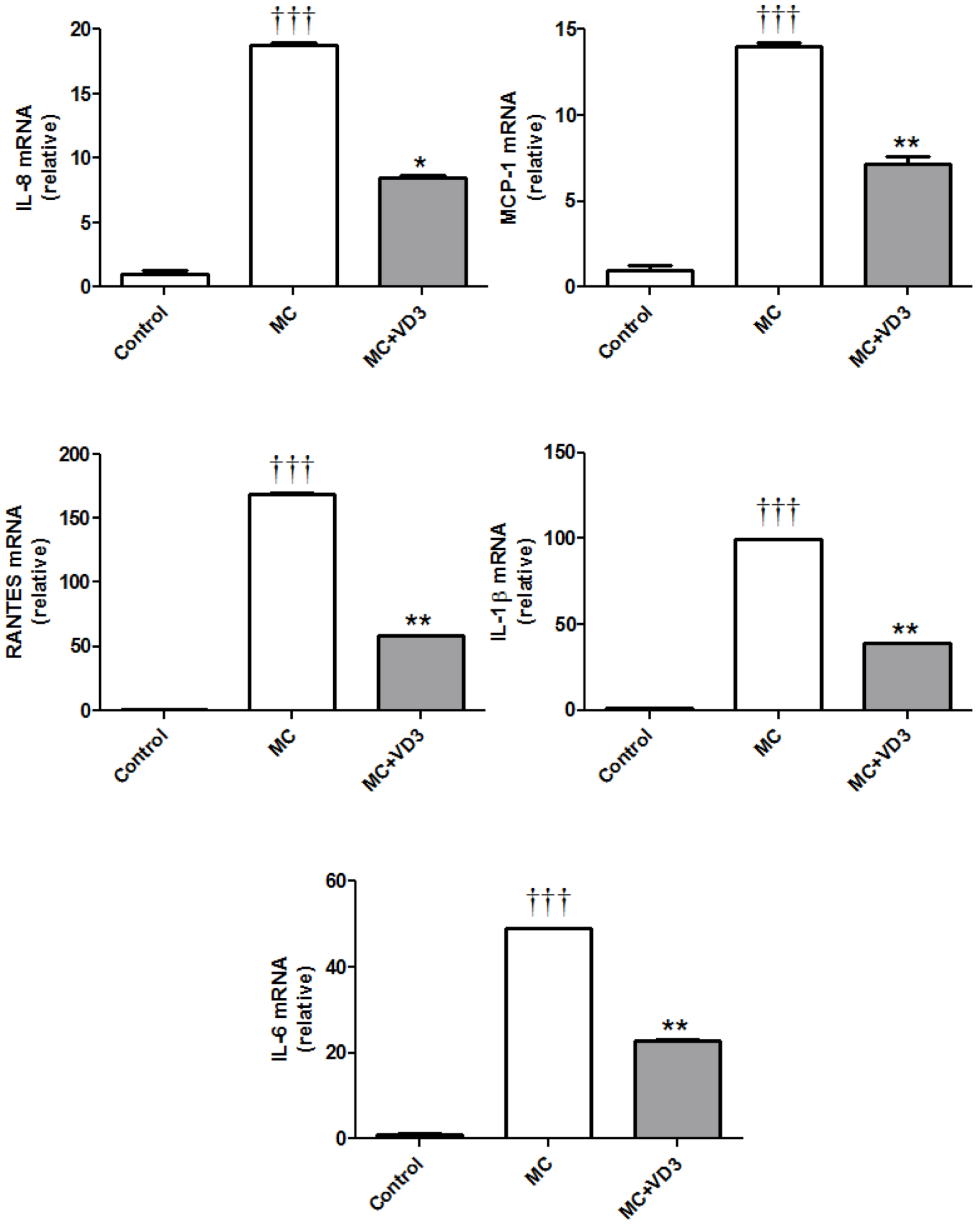
Effects of 1,25-dihydroxyvitamin D_3_ on MC medium-induced expression of the chemokines/cytokines by human adipocytes. Adipocytes were pretreated with 1,25(OH)_2_D_3_ (10^−8^ M) or without for 48 h, followed by the incubation with RPMI-1640 medium (control) or macrophage conditioned (MC) medium (25%) for another 4 h. mRNA levels of IL-8, MCP-1, RANTES, IL-1β and IL-6 were measured by real-time PCR and normalised to β-actin. Data are means ± SEM, n = 6 per group. ^†††^ *P*<0.001 vs controls; *P<0.05, **P<0.01 vs MC group. The results were verified by three independent experiments.

Since vitamin D_3_ inhibits the activation of the NFκB and MAPK signalling pathways, we further examined the downstream effect of vitamin D*_3_* on the gene expression and protein release of the proinflammatory cytokines/chemokines by adipocytes. As shown in Figure 6, exposure to MC medium (25%) for 4 h markedly increased mRNA levels of IL-8 (19-fold, *P*<0.001), MCP-1 (14-fold, *P*<0.001), RANTES (169-fold, *P*<0.001), IL-1β (100-fold, *P*<0.001) and (IL-6 (49-fold, *P*<0.001), compared with controls. This upregulation was significantly decreased by the pretreatment with 1,25(OH)_2_D_3_ (10^−8^ M): IL-8 (55%, *P*  = 0.013), MCP-1 (49%, *P*  = 0.008), RANTES (65%, *P*<0.004), IL-1β (61%, *P*  = 0.003) and IL-6 (53%, *P*  = 0.001).

**Figure 7:**
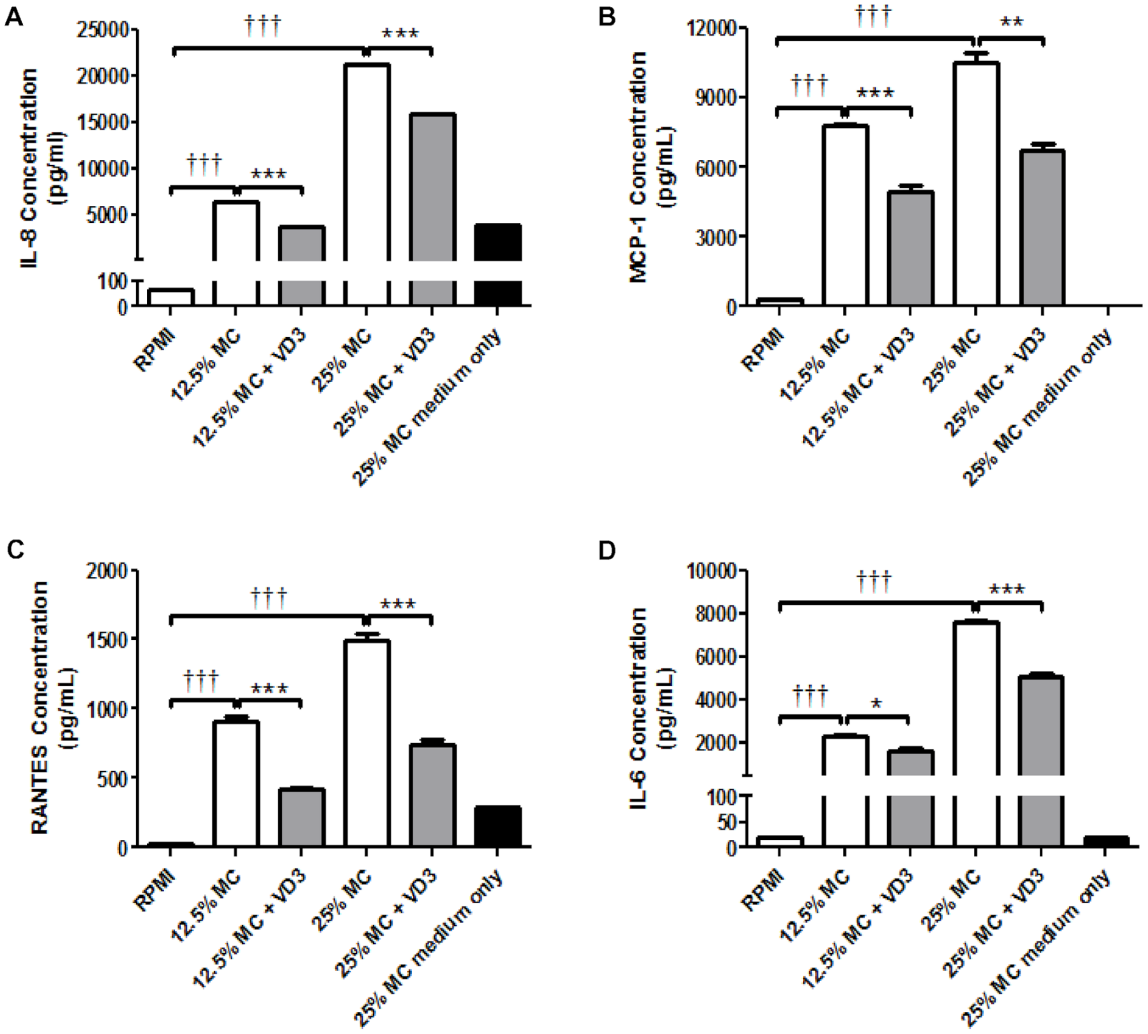
Effects of 1,25-dihydroxyvitamin D_3_ on MC medium-induced secretion of the chemokines/cytokine by human adipocytes. Adipocytes were pretreated with 1,25(OH)_2_D_3_ (10^−8^ M) or without for 48 h, followed by the incubation with RPMI-1640 medium (control) or macrophage conditioned (MC) medium (12.5% or 25%) for another 24 h. A separate group (25% MC medium only without cells) was included to show basal levels of chemokine/cytokines in the MC medium. Protein release of IL-8 (A), MCP-1 (B), RANTES (C) and IL-6 (D) was determined using ELISAs in supernatants. Data are means ± SEM, n = 6 per group. ^†††^ *P*<0.001 vs controls; *P<0.05, **P<0.01, ***P<0.001 vs MC group. The results were confirmed by three independent experiments.

Consistent with the mRNA results, in adipocytes exposed to MC medium (12.5% or 25%) for 24 h, there was a substantial and dose-dependent elevation in protein release of IL-8 (67-fold and 258-fold, both *P*<0.001), MCP-1 (27-fold and 34-fold, both *P*<0.001), RANTES (22-fold and 42-fold, both *P*<0.001) (Fig. 6D) IL-6 (111-fold and 368-fold, both *P*<0.001) (Fig. 6A–D). Treatment with 1,25(OH)_2_D_3_ (10^−8^ M) led to a significant reduction in MC medium-elicited release of IL-8 (61% and 31%, both *P*<0.001), MCP-1 (37%, *P*<0.001 and 36%, *P*  = 0.002) and RANTES (78% and 62%, both *P*<0.001) and IL-6 (29%, *P  = *0.019 and 34%, *P*<0.001) (Fig. 7A–D).

### 1,25-dihydroxyvitamin D_3_ Decreases Monocyte Migration

**Figure 8:**
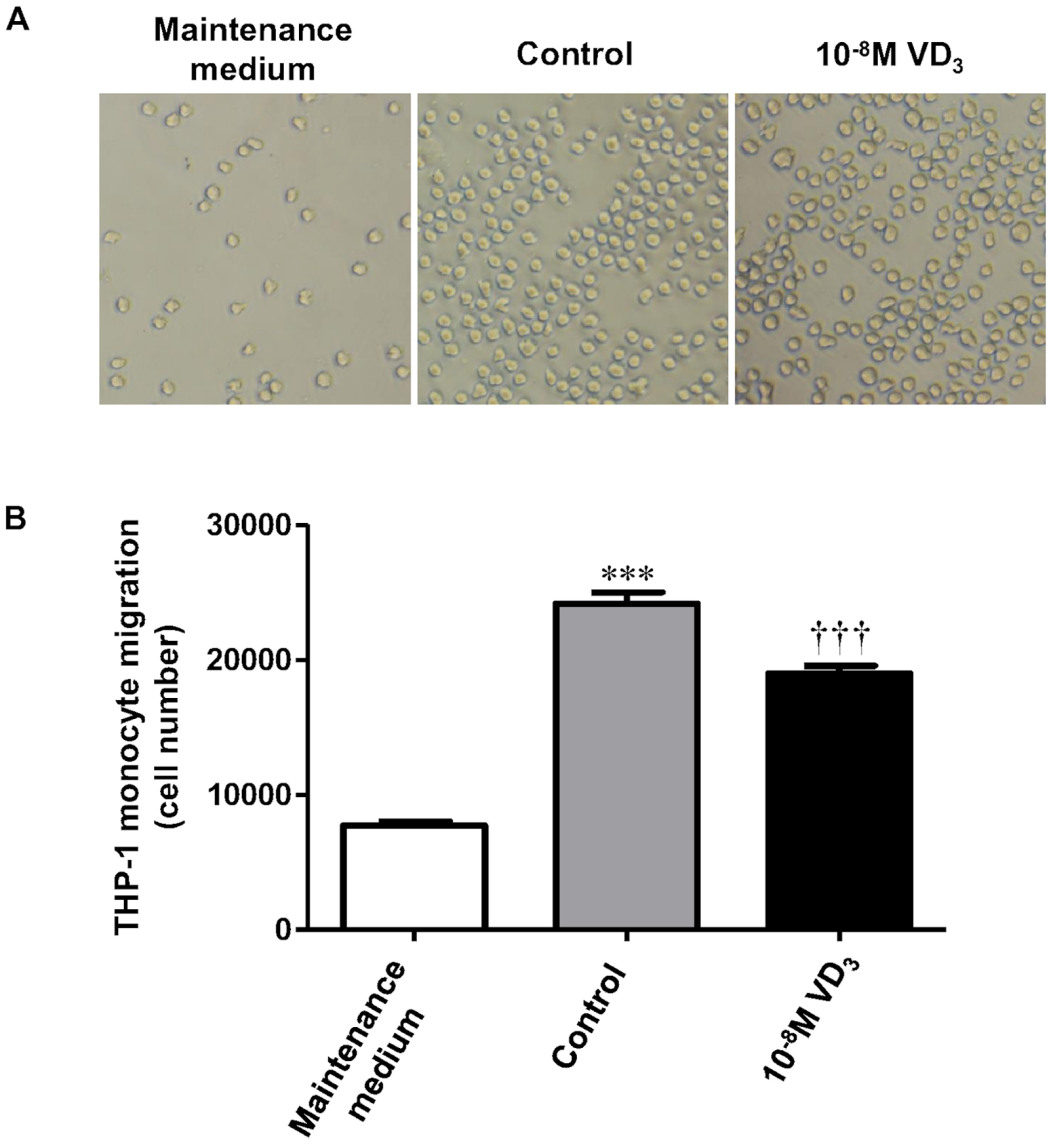
1,25-dihydroxyvitamin D_3_ reduces monocyte migration. Human adipocytes growing in maintenance medium were pretreated with 1,25(OH)_2_D_3_ (10^−8^ M) or without (control) for 24 h and the culture medium (150 µl) was harvested. The maintenance medium (without cells) (150 µl) was also collected. The medium was added to the lower chamber of the transwells and THP-1 monocytes (2×10^6^/ml; 100 µl) were placed to the upper chamber of the transwells. After incubation for 4 h at 37°C, the migration of monocytes was determined by the MTT assay. (A) Representative images of monocyte migration. (B) The number of monocytes migrated; data are means ± SEM, n = 3 per group. ***P<0.001 vs maintenance medium; ^†††^ *P*<0.001 vs controls. The results were confirmed by three independent experiments.

As vitamin D_3_ reduces the adipocyte production of the chemokines (i.e MCP-1, IL-8 and RANTES) which are known to have chemotactic effects, we then explored whether vitamin D_3_ affects chemotactic ability of adipocytes. This was determined as THP-1 monocyte migration induced by adipocytes pretreated with 1,25(OH)_2_D_3_ or without (control) for 24 h. As shown in Fig. 8A–B, The medium of adipocytes pretreated with1,25(OH)_2_D_3_ (10^−11^ and 10^−8^ M) resulted in a significant decrease in monocyte migration (by 25% and 21%, both *P*<0.001) compared with controls; maintenance medium alone (without cells) served as a negative control had the least effect on monocyte migration.

### MC Medium or 1,25-dihydroxyvitamin D_3_ Has No Cytotoxic Effect

Cell viability was assessed as LDH release by adipocytes and there were no significant differences between control (mean±SEM: 0.75±0.025) and treatment groups (10^−11 ^M VD_3_: 0.62±0.042; 10^−8 ^M VD_3_: 0.68±0.03; MC: 0.73±0.046; 10^−11 ^M VD_3_+ MC: 0.69±0.023; 10^−8 ^M VD_3_+ MC: 0.78±0.072) (all *P*>0.05). Therefore, MC medium or 1,25(OH)_2_D_3_ did not induce cytotoxicity.

## Discussion

In the present study, we used human THP-1 monocytes and human primary adipocytes as *in vitro* models to illustrate the inhibitory effects of 1,25(OH)_2_D_3_ on macrophage-induced inflammatory responses in adipocytes. We first examined whether 1,25(OH)_2_D_3_ prevents the activation of NFκB, which controls the transcription of proinflammatory cytokines in many cell types, including preadipocytes and adipocytes [21], [37], [38], [39]. NFκB activation is initiated by the degradation of IκBα protein, which allows the translocation of NFκB subunits into the nucleus thereby regulating downstream transcriptional programmes [38], [40]. In the present study we demonstrate that 1,25(OH)_2_D_3_ has a strong inhibitory effect on NFκB signalling in human adipocytes, as 1,25(OH)_2_D_3_ (10^−8^ M) increased basal IκBα levels and reversed inhibition of IκBα by the MC medium. Consistent with our data, recent studies have observed that in murine 3T3-L1 adipocytes, human preadipocytes and adipocytes, 1,25(OH)_2_D_3_ also increased protein abundance of IκBα [33], [34], [41]. Thus, 1,25(OH)_2_D_3_ could enhance the stability of IκBα to inhibit NFκB activation in adipocytes. In addition, we show that 1,25(OH)_2_D_3_ reduced basal and completely attenuated MC medium-induced phosphorylation of NFκB p65 in human adipocytes. NFκB p65 has been shown to be essential in the production of proinflammatory cytokines in human preadipocytes as NFκB p65 knockdown markedly reduced the release of IL-6 and IL-8 [20]. Recently,1,25(OH)_2_D_3_ (10^−7^ M) was shown to block NFκB p65 translocation to the nucleus in hMSC-derived adipocytes [41]. Taken together, these results suggest a role for 1,25(OH)_2_D_3_ in preventing the activation of NFκB signalling pathway in human adipocytes.

The signal transduction of inflammatory mediators may also involve the activation of the MAPK signalling. MAPK of the serine/threonine family, such as p38 MAPK, the extracellular signal-regulated kinases (Erk1/2) and the c-jun N-terminal kinase (JNK), contribute to the inflammatory response in various cell types [29], [42] although responses in human adipose tissue are largely unknown. We recently found that MAPK signalling is required in macrophage-induced increases in MMP1 and MMP3 production by human preadipocytes [21]. The release of MCP-1 from explants of human visceral adipose tissue was reduced by inhibitors of the p38 MAPK and NFkB pathways [43]. In the present study, we provide clear evidence that in human adipocytes, MC medium strongly induces phosphorylation of the p38 MAPK and Erk1/2 kinases. Of interest, the activation of MAPK signalling in omental fat in obese subjects has been suggested, as protein expression of phosphorylated p38 MAPK was increased by over 2-fold in obese women compared with lean controls and further, the expression level was positively correlated with clinical parameters such as plasma triglycerides and HOMA-IR (homeostasis model assessment for insulin resistance) [44]. We show in the present study that 1,25(OH)_2_D_3_ effectively decreased macrophage-induced phosphorylation of p38 MAPK in a dose-dependent manner. In addition to inhibiting p38 MAPK, 1,25(OH)_2_D_3_ also reduced basal, and totally abolished phosphorylation of Erk1/2 elicited by MC medium. Therefore, 1,25(OH)_2_D_3_ could act as a potent negative regulator of the MAPK signalling pathway in adipocytes, thereby blocking the transcriptional induction of proinflammatory factors. The molecular mechanisms by which vitamin D_3_ exerts effects on the signalling pathways remain to be established. Vitamin D_3_ acts by binding to its nuclear receptor VDR which then forms heterodimer with retinoid X receptors, and binds to vitamin D response elements (VDREs) located in promoter regions, thereby regulating the transcription of many target genes [45], [46]. In a recent study, vitamin D_3_ increased VDR binding to a putative VDRE in MKP-1 promoter and upregulated MKP-1 expression, which led to the inhibition of LPS-induced p38 MAPK phosphorylation and cytokine production in human blood monocytes [29]. VDR could be important in mediating the effects of 1,25(OH)_2_D_3_ on signalling pathways in human adipocytes and further studies are warranted.

Since 1,25(OH)_2_D_3_ inhibits the NFκB and MAPK pathways, we subsequently examined the downstream effects of 1,25(OH)_2_D_3_, particularly the production of the proinflammatory factors by adipocytes upon macrophage stimulation. We show that exposure of adipocytes to MC medium induced a striking increase in gene expression (14- to 169-fold) and protein release (22- to 368-fold) of the major chemokines/cytokines, including IL-8, MCP-1, RANTES, IL-1β and IL-6 (Fig.7). Our data suggests that macrophages are strong inducers of a proinflammatory state in adipocytes, which may form a positive autocrine/paracrine feedback circuit and also provide signals for recruiting macrophages and other immune cells. Additionally, chemokines (i.e. IL-8, MCP-1 and RANTES) are known to be produced at high levels by macrophages [22], which would cause further monocyte/macrophage accumulation in adipose tissue.

A key finding from the present study is the demonstration that 1,25(OH)_2_D_3_ powerfully inhibits MC medium-induced expression and release of the chemokines (IL-8, MCP-1 and RANTES) by human adipocytes. IL-8, a member of the CXC chemokine family, has significant chemotactic activity towards neutrophils [47]. In mice fed with a high-fat diet, there is a transient increase in neutrophil infiltration in intra-abdominal fat and IL-8 stimulates neutrophils adhered to 3T3-L1 adipocytes [48]. Other cell types including macrophages also respond to IL-8 as lack of IL-8 receptor CXCR2 protects from adipose macrophage recruitment and insulin resistance in diet-induced obese mice [49]. In severely obese subjects, circulating levels of IL-8 are increased [50]. IL-8 mRNA levels are upregulated in breast adipose tissue of obese women and this is in parallel with increased macrophage infiltration [51].We found that 1,25(OH)_2_D_3_ inhibited macrophage-induced IL-8 gene expression (by 53%) and release (up to 61%) from human adipocytes, suggesting vitamin D_3_ suppresses IL-8 production in adipose tissue.

MCP-1 (or CCL2) and RANTES (or CCL5) belong to CC chemokines which induce the migration of monocytes and other cell types [52]. MCP-1 and its receptor CCR2 are considered to be pivotal for macrophage infiltration in adipose tissue in obesity [53], [54]. In MCP-1 or CCR2 knockout mice, there is a decrease in macrophage infiltration in adipose tissue [54], [55] whereas overexpressing CCL2 enhances macrophage accumulation and insulin resistance [56]. The present study demonstrates an inhibitory effect of 1,25(OH)_2_D_3_ on MCP-1 expression and release by human adipocytes stimulated with MC medium. This is consistent with recent studies by our group and others that 1,25(OH)_2_D_3_ decreased MCP-1 secretion under stimulated (by TNFα, IL-1β and MC medium) conditions, in human preadipocytes and adipocytes [34], [35]. In addition to MCP-1, recent evidence suggests that RANTES is another key player in the inflammation of adipose tissue in obesity [57]. Serum levels of RANTES and its gene expression in adipose tissue are increased in obese subjects [58]. Furthermore, RANTES promotes monocyte transmigration and macrophage survival in human adipose tissue [58]. In contrast, blocking RANTES with a neutralising antibody reduced T-cell chemotaxis induced by media conditioned by adipose tissue of obese mice [59], and deletion of RANTES receptor CCR5 in mice protected against macrophage recruitment and M2- to M1-type adipose tissue macrophage (ATM) polarization [52]. However, whether vitamin D_3_ modulates RANTES production in human adipose tissue is not known. The present study reveals that 1,25(OH)_2_D_3_ strongly reduced the expression (by 66%) and release (up to 78%) of RANTES from human adipocytes upon macrophage stimulation. Moreover, we show that 1,25(OH)_2_D_3_ also inhibits adipocyte production of the major cytokines IL-1β and IL-6, both of which are critically involved in obesity associated inflammation and insulin resistance [60]. Although IL-1β and IL-6 do not possess chemotactic properties, indirect effects on monocyte recruitment for example via upregulation of chemokines cannot be excluded. A recent study from our group has reported that IL-1β provoked a large increase in MCP-1 release from human preadipocytes [34].

The vitamin D_3_ doses used in our study are based from physiological (i.e. 10^−11^ and 10^−10^ M) levels and pharmacological (i.e. 10^−9^ and 10^−8^ M) levels, which have been similarly employed in several published studies [29], [61], [62]. It should be mentioned that since adipocytes and macrophages are able to convert 25(OH)D_3_ to 1,25(OH)_2_D_3_ [14], [63], vitamin D_3_ concentrations in adipose tissue might be higher than circulating levels. Currently, data on the exact 1,25(OH)_2_D_3_ levels in human adipose tissue are scarce. In a small study of morbidly obese subjects (n = 17), 1,25(OH)_2_D_3_ concentrations determined by liquid chromatography–MS (LC/MS) were considerably higher (>10-fold) in subcutaneous fat than in serum [64]. Further studies are needed to reveal the levels of vitamin D_3_ in adipose tissue of lean and obese subjects.

**Figure 9:**
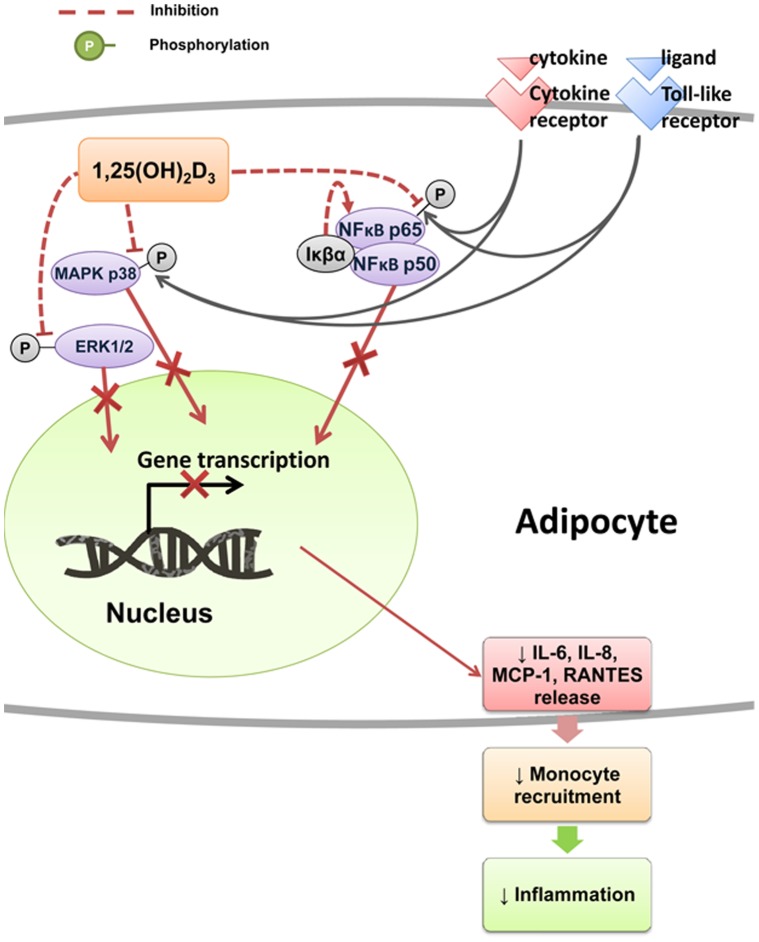
Schematic diagram of mechanisms of 1,25-dihydroxyvitamin D_3_ action in human adipocytes. 1,25(OH)_2_D_3_ has an inhibitory effect on the activation of the NFκB and MAPK signalling pathways, with increased IκBα expression while decreased phosphorylation of NFκB p65, p38 MAPK and Erk1/2. Consequently, there is a reduction in gene transcription and protein release of proinflammatory chemokines/cytokines, such as IL-8, MCP-1, RANTES and IL-6, by adipocytes, which may lead to reduced chemotaxis of monocytes/macrophages and adipose tissue inflammation.

Collectively, the results from the current study suggest that vitamin D_3_ is able to counteract the stimulatory effect of macrophages on the production of chemoattractants, such as IL-8, MCP-1 and RANTES, by adipocytes. As a result, this may disrupt the vicious cycle of perpetuating immune cell infiltration into adipose tissue. Consistent with this notion, we demonstrate that 1,25(OH)_2_D_3_ decreased the chemotactic ability of adipocytes since conditioned medium of adipocytes treated with 1,25(OH)_2_D_3_ (10^−11^ and 10^−8^ M) reduced monocyte migration (Fig.8). It is, therefore, probable that vitamin D_3_ acts favourably in adipose tissue to limit monocyte recruitment and its associated inflammation (Fig. 9).

In summary, we have shown that 1,25(OH)_2_D_3_ reduces macrophage-induced inflammatory responses in human adipocytes. 1,25(OH)_2_D_3_ strongly inhibits the activation of the NFκB and MAPK signalling pathways, which may prevent gene transcription of proinflammatory factors. Consistently, 1,25(OH)_2_D_3_ significantly decreases macrophage-elicited expression and release of the major proinflammatory chemokines/cytokines by human adipocytes. In addition, 1,25(OH)_2_D_3_ is able to reduce the chemotactic activity of adipocytes towards monocytes, probably as the result of lowered chemoattractant production. Overall these results suggest that vitamin D_3_ has an important role in adipocyte biology through its anti-inflammatory properties; this might be particularly beneficial when adipose tissue becomes inflamed in obesity.
